# A Case of Manic-Like Symptoms and Confusion After Supplement Ingestion

**DOI:** 10.7759/cureus.106949

**Published:** 2026-04-13

**Authors:** Payas Shah, Sydney Ehrman, Regina Reed

**Affiliations:** 1 Psychiatry, Reading Hospital Tower Health, West Reading, USA

**Keywords:** cognitive function, efficacy, herb-drug interactions, manic symptoms, pharmacokinetics, pharmacology, safety, supplement

## Abstract

A variety of etiologies can lead a patient to present with symptoms that appear manic. A wide differential should be considered, including delirium, which may in turn be caused by multiple etiologies. Substance ingestion, specifically of dietary supplements, is one potential trigger that is not always considered.

A 40-year-old male patient with a history of long-standing attention-deficit/hyperactivity disorder (ADHD), anxiety, and depression presented with anxiety, restlessness, memory gaps, nausea, low appetite, and insomnia. Prior to admission, the patient started taking an herbal supplement for 15 days and stopped taking lisdexamfetamine for 1.5 weeks. Upon initial presentation, the patient was hypertensive and tachycardic. The physical exam was significant for bilateral conjunctival injection and hand tremors. He was disoriented to time and had tangential, pressured speech. A comprehensive medical work-up was largely unremarkable. The urine drug screen was positive for amphetamines and marijuana. The Psychiatry Consultation and Liaison Service evaluated this patient and diagnosed him with delirium, possibly secondary to the supplement side effects. The supplement was discontinued, and the patient’s symptoms resolved within 24 hours.

While many diagnoses were considered for this patient, delirium, possibly triggered by starting a new herbal supplement, was determined to be the most likely etiology. Many supplements have not been well-studied and are inadequately regulated. This case highlights the potential for clinically significant adverse effects associated with supplement use and emphasizes the need for further research into their safety profiles, pharmacokinetics, and neuropsychiatric consequences to improve patient care and education.

## Introduction

Today, the majority of US adults report regular use of dietary supplements, and sales of dietary supplements comprise a multi-billion-dollar global industry [1]. Despite their wide consumption, dietary supplements are poorly regulated in the United States, and manufacturers are not required to provide evidence of product safety and efficacy. Conversely, medications are subject to strict pharmaceutical regulation by the Food and Drug Administration before being sold and marketed in the community [2]. Supplements are often marketed as “all-natural” products, and there is a common misconception that dietary supplements are equivalent to food and therefore do not interact with medications [3]. It is important to understand that supplements, on their own or when combined with medications, can have various side effects, including neuropsychiatric and behavioral changes [3]. For example, supplements may cause side effects that can be mistaken for mania. 

Manic episodes, as seen in bipolar disorder, are defined as a persistent elevated or irritable mood with increased activity or energy lasting for at least seven consecutive days or requiring hospitalization. Additionally, the presence of three or more associated symptoms (four if the mood is primarily irritable) is required to meet diagnostic criteria for a manic episode, including inflated self-esteem or grandiosity, decreased need for sleep, a compulsion to keep talking or being more talkative than usual, flight of ideas or racing thoughts, high distractibility, increased goal-directed activity or psychomotor agitation, and excessive involvement in risky behaviors that have negative consequences such as shopping sprees. The episode cannot be due to substance use or general medication conditions [4].

Bipolar disorder can present similarly to many other illnesses, including substance intoxication or medication side effects, attention-deficit/hyperactivity disorder (ADHD), and delirium. Symptoms of hyperactivity, distractibility, disinhibition, restlessness, racing thoughts, rapid speech, talkativeness, and irritability that are commonly seen in ADHD can overlap with manic symptoms. Delirium is a condition characterized by acute disturbances in attention, sleep-wake cycle, agitation, and cognitive impairment often precipitated by substance ingestion or withdrawal [4]. A contributor to such presentations may be the use of dietary supplements.

This case report describes a patient who developed acute neuropsychiatric symptoms after ingesting a supplement, emphasizing the importance of considering supplement-induced effects in the differential diagnosis when evaluating patients presenting with manic or delirium-like symptoms.

This case report was previously presented as a poster presentation at the Tower Health Research Day on March 27, 2025, and the Pennsylvania Psychiatric Society (PaPS) Central Chapter Resident and Medical Student Poster Contest on May 14, 2025. 

## Case presentation

This patient was a 40-year-old male patient with a history of ADHD (diagnosed in childhood), anxiety, depression (diagnosed greater than five years prior to current presentation), erectile dysfunction (ED), a history of gastric bypass, cholecystectomy, and gastroesophageal reflux disease (GERD) who presented to the hospital with anxiety, confusion, restlessness, gaps in memory, and decreased sleep for four days after starting a new supplement about two weeks prior. He began taking the supplement with the intention of improving memory and focus, and denied consuming this supplement prior to then. He also denied experiencing any episodes of similar symptoms in the past. 

In addition, the patient’s prescribed medications included trazodone 150 mg nightly, lisdexamfetamine 40 mg daily, omeprazole 40 mg daily, and tadalafil 5 mg oral on-demand (PRN) for ED. He reported having been prescribed this medication regimen for years. About 1.5 weeks prior to presentation, he self-discontinued lisdexamfetamine. He also reported self-reducing his trazodone from 150 mg to 75 mg nightly, but it is not clear exactly when he started taking the lower dose.

Approximate timeline of events

More than five years prior to presentation, the patient was diagnosed with ADHD, anxiety, and depression. One year prior to presentation, the patient was prescribed lisdexamfetamine for ADHD symptoms and trazodone for sleep. About two weeks prior to presentation, the patient began taking an over-the-counter supplement. About 1.5 weeks prior to presentation, the patient self-discontinued lisdexamfetamine due to abdominal pain. About four days prior to presentation, the patient developed anxiety, confusion, restlessness, memory gaps, and decreased sleep. On the day of presentation, he presented to the hospital due to the persistence and worsening of these symptoms. The supplement was discontinued. 

On the day after the presentation, the patient’s symptoms resolved. The Psychiatry Consultation and Liaison team was consulted due to behavioral changes on initial presentation. The patient was diagnosed with delirium.

Upon initial admission to the hospital, the patient was hypertensive with a blood pressure of 154/95 mmHg, and his pulse ranged from 70 to 102 beats per minute; other vital signs were stable. Physical examination was notable for conjunctival injection and tremor in both hands. Initially, he was disoriented to time and displayed tangential, pressured speech. Due to agitation, he was treated with two doses of lorazepam 1 mg IV and olanzapine 5 mg. Following treatment, his mental status progressively improved, with complete resolution of altered mental status within 24 hours.

A comprehensive medical work-up was done, including a complete blood count, a comprehensive metabolic panel, a creatine kinase level, a Tylenol level, a salicylate level, a urinalysis, a urine drug screen, thyroid-stimulating hormone, a CT and MRI of the brain, a chest X-ray, a hepatitis panel, and a video electroencephalogram. The patient was found to have elevated creatine kinase at 778 IU/L, a potassium level of 3.2 mmol/L, aspartate aminotransferase of 48 IU/L, hemoglobin of 12.7 g/dL, and a urine drug screen positive for amphetamines and marijuana. All other labs and imaging were within normal limits. By the end of the hospitalization, the creatine kinase level stabilized to 508 IU/L with IV fluids, and all other lab abnormalities normalized (Tables 1-3).

**Table 1:** Serum Toxicology Labs

Lab Test	Reference Range	Result
Blood Alcohol Level	<10 mg/dL	< 3
Tylenol	10.0-20.0 mcg/mL	< 2
Salicylate Level	<30 mg/dL	< 3

**Table 2:** Qualitative Urine Drug Screen THC: tetrahydrocannabinol; PCP: phencyclidine

Lab Test	Result
Amphetamines	Positive
Barbiturates	Negative
Benzodiazepines	Negative
Cocaine	Negative
Opiates	Negative
THC (Marijuana)	Positive
PCP Screen, Urine	Negative
Methadone	Negative
Oxycodone	Negative
Fentanyl	Negative

**Table 3:** Supplemental Labs CO_2_: carbon dioxide; BUN: blood urea nitrogen; eGFR (CKD-EPI): estimated Glomerular Filtration Rate (Chronic Kidney Disease Epidemiology Collaboration); eGFR (MDRD): estimated Glomerular Filtration Rate (Modification of Diet in Renal Disease); AST: aspartate aminotransferase; ALT: alanine transaminase; WBC: white blood cell count; RBC: red blood cell count; MCV: mean corpuscular volume; MCH: mean corpuscular hemoglobin; MCHC: mean corpuscular hemoglobin concentration

Lab Test	Reference Range	Day 1 Result	Day 2 Result
Sodium	136-145 mmol/L	139 mmol/L	143 mmol/L
Potassium	3.5-5.1 mmol/L	3.2 mmol/L	3.5 mmol/L
Chloride	98-107 mmol/L	106 mmol/L	112 mmol/L
CO_2_	20.0-31.0 mmol/L	26.0 mmol/L	25.0 mmol/L
Glucose	74-99 mg/dL	118 mg/dL	124 mg/dL
BUN	9-23 mg/dL	11 mg/dL	8 mg/dL
Creatinine	0.73-1.18 mg/dL	1.04 mg/dL	0.87 mg/dL
Calcium	8.7-10.4 mg/dL	10.0 mg/dL	8.5 mg/dL
eGFR (CKD-EPI)	>60 mL/min/1.73m^2	93.0 mL/min/1.73m^2	111.8 mL/min/1.73m^2
eGFR (MDRD)	-	79.10	97.19
Anion Gap	5-12 mmol/K	6 mmol/K	6 mmol/K
AST	<34 IU/L	48 IU/L	31 IU/L
ALT	10-49 IU/L	26 IU/L	17 IU/L
Bilirubin Total	0.3-1.2 mg/dL	0.5 mg/dL	0.4 mg/dL
Bilirubin Direct	<=0.3 mg/dL	0.2 mg/dL	Not Collected
WBC	4.8-10.8 10E3/uL	6.4 10E3/uL	5.2 10E3/uL
RBC	4.50-6.10 10E6.uL	5.14 10E6.uL	4.50 10E6.uL
Hemoglobin	14.0-17.5 g/dL	12.7 g/dL	11.1 g/dL
Hematocrit	39.0-53.0%	40.2%	36.2%
MCV	80.0-99.0 fL	78.2 fL	80.4 fL
MCH	27.0-34.0 pg	24.7 pg	24.7 pg
MCHC	31.0-37.0 g/dL	31.6 g/dL	30.7 g/dL
Platelets	130-400 10E3/uL	309 10E3/uL	240 10E3/uL
Creatine Kinase	46-171 IU/L	508 IU/L	Not Collected

Psychiatry was consulted due to behavioral changes in the setting of self-discontinuation of home medications, and he was seen by the Psychiatry Consultation and Liaison service one day after admission. Upon psychiatric evaluation, the patient reported that he was in his usual state of health until four days prior to arrival at the hospital. He endorsed decreased sleep, anxiety, decreased appetite, restlessness, and occasional nausea. The patient reported that he had slept a total of three hours over four days. He stated that the day before admission, he felt lightheaded, jittery, and clumsy, and had increased energy, muscle cramping, and gaps in memory. He also stopped taking lisdexamfetamine about two weeks prior to admission due to abdominal pain. At the time of the interview, the patient reported that he felt “spacey,” but all other symptoms had resolved. The patient denied any prior symptoms of post-traumatic stress disorder, psychosis, or mania. On the mental status exam, he was alert, oriented, and had a linear and logical thought process with no abnormalities in thought content or perception. He denied homicidal and suicidal ideation. His appearance, behaviors, speech, and affect were appropriate, and his insight and judgment were fair.

The patient’s social history was significant for marijuana and caffeine use, and he denied using other substances. He reported that he smoked marijuana two to three times per week for many years. He also reported that he drank about two caffeinated energy beverages daily for many years. The patient lived with his significant other, who also confirmed the history provided by the patient.

The Psychiatry Consultation and Liaison Service diagnosed the patient with delirium. The differential diagnosis for delirium in this case included supplement side effects, and thus, the consultant's recommendation was to discontinue the supplement. Psychiatry also recommended that the patient continue his psychiatric medications, including lisdexamfetamine 40 mg and trazodone 75 mg, which he was taking previously.

## Discussion

This report summarizes the case of a 40-year-old male patient who presented with symptoms reminiscent of mania. These symptoms resolved or improved over the course of less than 24 hours. It is important to consider the wide differential diagnoses that exist for the symptoms experienced by this patient, including exacerbation of ADHD, acute manic episode, substance intoxication, delirium, supplement side effects, and various other medical conditions.

One potential cause of this patient’s symptoms is the exacerbation of ADHD. Of note, his lisdexamfetamine dose reportedly had been reduced and then self-discontinued prior to admission. His symptoms of increased energy and restlessness could be consistent with ADHD exacerbation. However, ADHD does not typically cause memory impairments, disorientation, or tangential thought processes [4], which were among our patient’s presenting symptoms. Furthermore, our patient reported reducing his dose or stopping his medication multiple times in the past without experiencing symptoms similar to the current presenting symptoms.

Another potential cause of this patient’s symptoms is bipolar mania, which is consistent with reported increased energy, restlessness, and decreased sleep for four days. However, it should also be noted that this patient had no prior history of manic symptoms and has never taken medication for bipolar disorder. He was never psychiatrically hospitalized, and symptoms resolved in a time frame that is faster than expected for a manic episode. Therefore, bipolar mania was lower on the differential diagnosis.

The case could be made that the patient’s symptoms are due to substances. The patient reports daily use of caffeinated beverages and regular cannabis use. Caffeine intoxication can induce symptoms reminiscent of mania, including insomnia, excitement, nervousness, restlessness, psychomotor agitation, and flight of ideas, all of which overlap with clinical features of manic episodes [5]. However, the patient reported consistent use of two caffeinated beverages daily for many years. He also reported that his cannabis use has been consistent for many years. The stability of the patient’s use of caffeine and cannabis makes it unlikely that these substances contributed to the patient’s presenting symptoms. Another substance to consider is amphetamines. The patient reported self-discontinuing lisdexamfetamine 1.5 weeks prior to presentation. However, the urine drug screen collected upon admission was positive for amphetamines. Nevertheless, there is no documented history of methamphetamine use in the chart; the patient denied using methamphetamines, and his history is corroborated by his partner. Additionally, it is important to mention that the patient was taking trazodone, which can result in false positives for amphetamines in the urine drug screen [6]. While methamphetamines cannot be definitively ruled out as a possible etiology, the above factors decrease the likelihood of methamphetamine use contributing to observed symptoms.

Lastly, delirium may account for the patient’s symptoms. The acute onset of symptoms, combined with fluctuating levels of consciousness, disorientation, and memory deficit, makes delirium the most likely explanation for the patient’s symptoms. A variety of etiological factors may contribute to the development of delirium, including infectious, metabolic, substance withdrawal, and acute intoxication. The patient did not have any laboratory abnormalities consistent with a major metabolic disturbance, infection, or an alternative medical cause that would explain his acute symptomatology. This patient stopped taking 40 mg lisdexamfetamine for a week and a half prior to admission due to abdominal pain, raising the possibility of withdrawal. However, withdrawal symptoms from lisdexamfetamine would be expected to include hypersomnolence and hyperphagia rather than increased energy, restlessness, and decreased sleep [4]. When considering intoxication as a cause of delirium, often the first things that come to mind are drugs and toxins. However, supplements should also be considered. This patient’s symptoms appeared shortly after he started taking a commercially available supplement. Once the supplement was discontinued, the symptoms resolved, and the patient was able to be discharged from the hospital after one day. This temporal relationship is suggestive of the supplement contributing to the patient’s presentation.

A WHO-Uppsala Monitoring Centre (UMC) causality assessment, a tool used to classify relationship likelihood in case reports, was completed. In this case, the patient’s symptoms developed within a reasonable amount of time following supplement ingestion and resolved following withdrawal of the supplement. However, alternative causes of the clinical presentation could not be definitively excluded. Given the above, restarting the supplement to evaluate for recurrence of symptoms was not appropriate, therefore supporting that the most appropriate causality category is “possible” [7].

Several popular supplements have been associated with delirium or other psychotropic adverse effects, including St. John's wort, ginseng, ephedra, and kava. St. John’s wort is used to improve mood, energy, concentration, and sense of well-being, but case reports have shown it can also cause fatigue, dizziness, headache, restlessness, irritability, insomnia, pressured speech, hypersexuality, and psychotic symptoms such as ideas of reference, auditory hallucinations, paranoid delusions, and loosening of association [8]. Case reports on ginseng, marketed to relieve fatigue and stress, describe use resulting in increased energy, decreased sleep, grandiosity, and agitation [8]. Another herbal supplement, *Ephedra*, has been marketed as a weight loss and exercise enhancement supplement. It acts as a stimulant in the central nervous system, producing a combination of dopaminergic and adrenergic effects resulting in improved mood, heightened awareness, decreased fatigue, and insomnia. At higher doses, it can cause restlessness and anxiety, and with prolonged use, it can be neurotoxic and lead to psychosis, severe depression, mania, agitation, hallucinations, sleep disturbances, and suicidal ideation [9].  Lastly, kava is marketed for its calming effects in patients with anxiety, but has also been shown to cause altered mental status and ataxia in some patients [10].

While these herbal supplements have been studied with respect to adverse effects, the same cannot be said about many other supplements, which may unknowingly cause adverse effects such as delirium. The supplement that this patient was consuming did not contain the above ingredients but did contain the following: vitamin B6 (pyridoxine HCl), L-tyrosine, L-theanine, oat (straw) extract, phosphatidylserine, cat’s claw (bark) extract, L-alpha glycerylphosphorylcholine (alpha-GPC), Bacopa (leaves) extract, toothed clubmoss (whole) extract (huperzine A), L-leucine, and pterostilbene.

Cat’s claw (bark) extract, a plant from the Amazon rainforest, is marketed for its anti-inflammatory and analgesic effects for the treatment of muscle and joint pain, fever, fatigue, and other chronic inflammatory conditions. However, there are no conclusive scientific evidence-based studies that support the use of this ingredient for health purposes [10]. The known side effects include nausea, diarrhea, gastrointestinal upset, headache, dizziness, and vomiting [11].

Bacopa (leaves) extract, a creeping perennial plant described as a “calming cognitive enhancer," is marketed for memory improvement, epilepsy, insomnia, and as an anxiolytic [12]. The most common side effects of Bacopa (leaves) extract are gastrointestinal side effects, including nausea, increased stool frequency, and abdominal cramps, which are secondary to an upregulation of acetylcholine activity. The cholinergic properties of Bacopa may increase the likelihood of side effects from other cholinergic drugs due to an additive effect or decrease the effectiveness of anticholinergic medications [13]. Bacopa has not been shown to be effective for any disease or medical symptom in controlled, prospective trials in humans and is not approved in the United States as therapy for any medical condition [11].

Toothed clubmoss (whole) extract (standardized to 1% huperzine A, 400 mcg) contains small amounts of huperzine A, which is marketed for the treatment of neurodegenerative diseases, including schizophrenia, cognitive dysfunction, and dementia. This ingredient is a competitive reversible inhibitor of acetylcholinesterase and may produce cholinergic side effects, including vomiting, dizziness, headache, restlessness, excitement, fatigue, and diarrhea [14].

Pterostilbene is marketed as an agent with anti-inflammatory, antioxidant, and anti-tumor properties. Unfortunately, much is not known about this ingredient, as research articles have noted that while preliminary clinical studies are encouraging, more rigorous trials are imperative to understand the safety and therapeutic benefits. It is reported that this ingredient does not cause severe side effects, but it is unknown what side effects are being studied and measured. Additionally, long-term safety data are lacking, and some studies have concerns about potential liver toxicity [15].

Oat (straw) extract is marketed for psychotropic indications, including improved cognitive function in terms of working memory and dual task performance. One study reported that side effects were “minor” but did not list what side effects were experienced by participants [16].

Lastly, the supplement taken by the patient also includes L-tyrosine, L-leucine, L-theanine, vitamin B6, L-alpha GPC, and phosphatidylserine. There is limited data about the safety profile of some of these supplement ingredients and a lack of substantial evidence of beneficial use in the literature, with the exception of vitamin B6 for deficiency states.

While individually the above supplements may cause minor side effects, it is important to consider the additive effects of these ingredients and also potential interactions with prescribed medications. In addition to the side effects of herbal supplements, the pharmacokinetic and pharmacodynamic properties of drug-herb interactions must be kept in mind. Pharmacokinetic interactions between an herbal supplement and a drug can occur when the herbal supplement shares the same mechanisms of absorption, distribution, metabolism, and excretion as the drug. As a result, the drug’s concentration at the site of action may be altered. Additionally, drug-herb interactions may result in pharmacodynamic interactions, which can occur when an herbal supplement directly affects the mechanism of action of the drug, unrelated to the drug’s concentration [17]. For example, cat’s claw can cause herb-drug interactions, as it is an inhibitor of the CYP3A4 enzyme, therefore increasing the concentration of drugs that are metabolized by this enzyme [6]. Additionally, Bacopa (leaves) extract has an inhibitory effect on CYP3A4. This is relevant to the current patient, as he was taking trazodone, which is a substrate for CYP3A4. Physicians should be aware of this ingredient as it has a major impact on many CYP isoforms, including CYP2C9, CYP2C19, CYP1A2, CYP2D6, and CYP3A4 [18]. It is crucial to consult pharmacists or herbal specialists to assess the safety of certain herbal supplements and the potential drug-herb interactions that may be at play.

There are several limitations of this study. First, as with all descriptive studies, it is not possible to prove a cause-and-effect relationship. Second, this is a case report on a single patient and is therefore not a representative population. Consequently, it is not possible to generalize the findings. Third, much of the relevant data was obtained retrospectively using the patient’s electronic medical record, making the study findings reliant upon the accuracy and completeness of the documentation. Finally, it is not possible to test the supplement for potential contaminants or rule out all possible cofounders that may have contributed to the patient’s presentation.

## Conclusions

This case study highlights the importance of conducting a comprehensive medication reconciliation with patients that includes home medications as well as dietary supplements, herbal supplements, and other complementary medications. It is critical to obtain collateral information from family members or other providers, such as herbalists, to inquire about supplements. Further, understanding the mechanism of action of supplements is important when considering drug-herb interactions. There may be benefits associated with the use of herbal supplements for certain conditions; however, many supplements can cause side effects or interactions with medications that are dangerous for patients. Given the widespread use of supplements, improved regulatory oversight and post-marketing surveillance of supplements are necessary to better identify potential safety concerns. In light of their popularity, increasing literature on both the benefits and risks associated with herbal supplements would allow consumers and providers to make informed choices.
